# Neurophysiological Responses to Different Product Experiences

**DOI:** 10.1155/2018/9616301

**Published:** 2018-09-24

**Authors:** Enrica Modica, Giulia Cartocci, Dario Rossi, Ana C. Martinez Levy, Patrizia Cherubino, Anton Giulio Maglione, Gianluca Di Flumeri, Marco Mancini, Marco Montanari, Davide Perrotta, Paolo Di Feo, Alessia Vozzi, Vincenzo Ronca, Pietro Aricò, Fabio Babiloni

**Affiliations:** ^1^Departmentof Molecular Medicine, Sapienza University of Rome, Viale Regina Elena 291, 00161 Rome, Italy; ^2^Departmentof Anatomical, Histological, Forensic & Orthopedic Sciences, Sapienza University of Rome, Piazzale Aldo Moro 5, 00185 Rome, Italy; ^3^Department of Communication, Social Research, Sapienza University of Rome, Via Salaria 113, 00198 Rome, Italy; ^4^IRCCS Fondazione Santa Lucia, Neuroelectrical Imaging and BCI Lab, Via Ardeatina 306, 00179 Rome, Italy; ^5^BrainSigns Srl, Rome, Italy; ^6^Department of Computer Science, Hangzhou Dianzi University, Xiasha Higher Education Zone, 310018 Hangzhou, China

## Abstract

It is well known that the evaluation of a product from the shelf considers the simultaneous cerebral and emotional evaluation of the different qualities of the product such as its colour, the eventual images shown, and the envelope's texture (hereafter all included in the term “product experience”). However, the measurement of cerebral and emotional reactions during the interaction with food products has not been investigated in depth in specialized literature. The aim of this paper was to investigate such reactions by the EEG and the autonomic activities, as elicited by the cross-sensory interaction (sight and touch) across several different products. In addition, we investigated whether (i) the brand (Major Brand or Private Label), (ii) the familiarity (Foreign or Local Brand), and (iii) the hedonic value of products (Comfort Food or Daily Food) influenced the reaction of a group of volunteers during their interaction with the products. Results showed statistically significantly higher tendency of cerebral approach (as indexed by EEG frontal alpha asymmetry) in response to comfort food during the visual exploration and the visual and tactile exploration phases. Furthermore, for the same index, a higher tendency of approach has been found toward foreign food products in comparison with local food products during the visual and tactile exploration phase. Finally, the same comparison performed on a different index (EEG frontal theta) showed higher mental effort during the interaction with foreign products during the visual exploration and the visual and tactile exploration phases. Results from the present study could deepen the knowledge on the neurophysiological response to food products characterized by different nature in terms of hedonic value familiarity; moreover, they could have implications for food marketers and finally lead to further study on how people make food choices through the interactions with their commercial envelope.

## 1. Introduction

During a shopping experience in a supermarket, it has been suggested for consumers to have few seconds to get in touch with the product in the aisle and to decide whether buying or not the product on the basis of the gathered information in that time window [1].

There have been many studies which have demonstrated the role and the importance of packaging in terms of ability to communicate relevant product information, its influence on consumers' attention, perception, and purchase intention [2,3]. Expressions such as “the silent salesman” [4] are commonly used to describe the role of packaging. Packaging has become a significant marketing channel because of its presence in the shop, combined with its strong influence on customers' decisions [5].

More recently, there has been a growing interest surrounding the influence of the sensory characteristics of packaging on consumers' expectations and on consumers' subsequent food experience [6, 7]. In fact, the product could be perceived as a combination of different items: the package, the brand, the aesthetic side (color, graphic, images, and shape), and the context of usage [8]. Each of these items may elicit different cognitive and emotional reactions with different meanings for consumers. Several studies have demonstrated that the role of the packaging is as important at the moment of the purchase as during the phase of usage and usability of product, known as the first and the second moment of truth, respectively [9,10]. The first one deals with the package's ability to grab the attention of customers, when the consumer builds his own expectations; this moment corresponds to the first time he looks at it, approaches, and deals with it. The second one is the experience itself, when the consumer uses and consumes the product. This is the moment of truth for the design of the prerequisites for services (i.e., information and functions). The package must be easy to use, the information on it must be relevant, so that consumers do not misuse the product, it must fit in storage spaces, and if the product should be dosed, the package has to facilitate this, and so on. Then, it is crucial for companies to design packages with user-friendly prerequisites for services since there are no employees present during the service consumption process. One could say that the package bridges the gap between production and consumption. If the visual exploration of food package provides information on the aesthetic side, the somatosensory inflow derived from its direct tactile exploration provides important information to the consumer [11]. In fact, touch gathers various data from perception, such as pressure, temperature, and texture [12], and it has a link to arousal [13]. Furthermore, arousal is the basis of emotion, motivation, and behavioural reactions; studies have shown that 95% of thinking is unconsciously realized where consumers make purchasing decisions based on emotion rather than rational thoughts [14]. Hurley's studies [15] showed that electrodermal activity, which in general is accepted as one of the more effective biomarkers of human arousal [16], is a possible measure to provide insight into customer preference of packaging and a support of a more holistic understanding of decision making. The correlation between vision and touch plays a very important role because the consumer develops a “feel” for the food package. In addition, the evaluation of the ingredients inside [17] would evoke memories of the food's taste and bring the somatic markers that were stored beside such memories back to the body. It is thus crucial in understanding consumer behaviour during evaluations of products, as studied in the literature, to study such variation of cerebral and emotional state of the consumers [18].

For years, the companies invested in traditional marketing researches, for example, surveys and focus groups, asking people to explain their consumption behaviour or to rate their preferences. Part of the recent growth on interest in multisensory packaging design is undoubtedly due to the potential use of the innovative techniques in the field of cognitive neuroscience, which allow to deeply understand the cognitive perception toward stimuli. Most attention has been given to pricing, products, and branding, which have demonstrated some of the benefits by using neuroscientific methods, specifically fMRI, during a shopping task. Those studies found that adding the information of neural measures to self-report led to significantly more accurate predictions of subsequent purchasing decisions. Furthermore, Plassman and colleagues used fMRI to study whether information that creates expectations about product quality can influence product perceptions [19]. Results of this investigation found support for the latter, demonstrating that changing the prices of otherwise identical products (wines) affected brain regions involved in interpreting taste pleasantness after tasting the wines [17]. Neuroscientific techniques have been used to better understand consumer behaviour and nonconscious interactions with consumer products, and the role of emotions in the decision-making process [20, 21]. These techniques include the measure of electroencephalographic signals (EEG), the brain imaging (e.g., fMRI), and the measure of autonomic nervous system measures, for example, heart rate (HR) and electrodermal response (EDR). EEG is a noninvasive method of recording the electrical activity of the brain, by placing electrodes on the scalp. EEG-based studies, for instance, investigated the perceived pleasantness toward a stimulus, demonstrating the existence of an asymmetric activity of the prefrontal cortex in the alpha band [22], which implies a different motivational tendency toward the proposed stimuli. In particular, various studies evidenced a relative increase in the left prefrontal cortex (PFC) activation in the duration of the positive motivation, while an augmented right-sided anterior activation during the negative motivation [23]. From these considerations, it is possible to define the Approach Withdrawal Index (AW) as the difference of the brain activity between the right and the left PFC in the alpha band. The positive value of the AW Index suggests a positive motivation toward the stimulus, while higher right activity would be reflected by negative frontal alpha asymmetry values, suggesting a negative motivation in response to the stimulus [24].

It has been evidenced how the higher values of EEG spectral power over the frontal scalp areas in theta frequency band (4–7 Hz) have been connected to higher levels of task difficulty [25]. It has been also suggested that such increase of EEG frontal midline theta power spectra values (hereafter “frontal theta” for brevity) can represent a marker of cognitive processing, occurring in correspondence of mental fatigue during different visuocognitive task [26]. The frontal theta, used as index of effort and processing in regard to the task's complexity, has already been applied in different research fields: neuroaesthetics field [27]; avionic and car driving for the detection of the effort employed in the execution of flight simulation, air traffic management, and driving tasks [25–32]; and different challenging listening conditions both in normal hearing than in hearing-impaired participants [33] and to human-computer interaction [34] studies.

Similarly to the measurement of EEG activity, the measure of the autonomic nervous system's (ANS) activity can help researchers understand the psychophysiological reactions to stimuli, which have been associated with emotional processes in academic literature [35, 36]. For the emotions, discrete and dimensional theoretical models have been designed, respectively, implying for emotional states: peculiar physiological, behavioural, and experiential correlate with the discrete models, while a combination of affective dimensions, mainly valence and arousal, correlates with the dimensional models [13]. With the purpose of estimating the emotional reaction to stimuli, for this work, beyond EEG measures, we recorded the electrodermal response (EDR) and heart rate (HR). The first one is used to measure electrodermal activity, a property of the human body that causes continuous variation in the electrical characteristics across the skin. Skin conductance (SC) varies with the activity of sweat glands in the skin (the higher the sweating is, the higher the SC will be), controlled by the sympathetic nervous system; therefore, skin conductance is an indicator of psychological or physiological arousal [37]. In the present research, an autonomic index has been adopted, resulting from the combination of the electrodermal response (EDR) and the heart rate (HR). These two signals reflect the emotional response to stimuli, and the resulting Emotional Index (EI) has been conceived starting from Russell and Barrett's circumplex model of affect [38], where the HR is plotted on the *x*-axis, while the EDR is plotted on the *y*-axis, respectively, reflecting information concerning the stimuli' valence (positive or negative) and arousal (low or high activation).

In this way, it is possible to obtain a monodimensional variable, called the Emotional Index (EI), which provides information concerning the emotional status of participants, as shown in previous studies [39]. Studies demonstrated that knowledge in neuroscience can potentially enrich research in decision making and integrating neuroscience with decision making has a lot of potential [40].

According to these evidences, the aim of this paper was to investigate the cognitive and emotional reactions to the cross-sensory interaction (sight and touch) with products belonging to different categories. We estimated such cognitive and emotional reactions by using the Mental Effort, the AW, and the Emotional Indexes mentioned above. In particular, we investigated the influence of the following three features of the products:

1. The brand: Major Brand or Private Label
2. The familiarity: Foreign or Local Brand
3. The hedonic value of the products: Comfort Food or Daily Food

First, two phases have been investigated: visual exploration (VE) and visual and tactile exploration (VTE) during the interaction with (i) two Daily Food items (one representative of a Major Brand and one representative of a Private Label) and (ii) two Comfort Food items (one representative of a Foreign product and one of a Local product) (Experiment 1). Furthermore, we extended the research to the study of the interaction between the brand and the familiarity by testing Comfort Food and Daily Food belonging to Major Brand, Private Label, and Foreign and Local products categories. Finally, in order to investigate the contribution of different sensory modalities, we added the tactile exploration (TE) phase to the already adopted VE and VTE phases (Experiment 2).

We made three working hypotheses:

*HP1*: Comfort Foods are a category of food, often characterized by high sugar and/or carbohydrate content, that should provide some psychological and specifically emotional reward (for a review, see [41]). Furthermore, since the prefrontal cortex (PFC) has been linked to the processing of hedonic aspects of taste [42], we predict higher AW values in response to the interaction with the Comfort Food than with the Daily Food. Furthermore, evidence shows that one sensory modality could be via by which the hedonic attributes of a product can be perceived and could bias the estimation of the quality and pleasantness of the product acquired by other sensory modalities [43]. Therefore, we would try to elucidate whether the visual or the tactile sensory modality would equally modulate the reaction (reflected by a similar pattern reported in both the VE and TE phases) or whether one of those would be predominant (higher values in one of the phases), or finally if they exerted a synergic effect (higher values in the VTE phase, but not in the TE and VE phases).

*HP2:* since (i) the higher complexity of a stimulus requests higher information processing, producing increased mental effort [25] and (ii) the increased tendency of approach, elicited by complex stimuli, raises the individual's interest [44], we hypothesize that the values of the investigated Cerebral Indexes for the participants would be higher during the interaction with Foreign products than with Local ones. In particular, it would occur during the VE and VTE phases, but not for the TE phase, due to the unfamiliarity of the products and because of the labels not being written in the native language.

*HP3:* literature evidences showed a differential activation pattern in the frontal lobe in response to the exposure to attractive and unattractive stimuli [45]. Typically, Major Brand products present more attractive packaging than products; therefore, we hypothesize that the former would elicit higher approach tendency than the Private Label items.

## 2. Materials and Methods

The project involved 32 healthy volunteers. Informed consent was obtained from each participant after the explanation of the study, which was approved by the local institutional ethics committee. The experiment was conducted following the principles outlined in the Declaration of Helsinki of 1975, as revised in 2000. During the entire experiment, the brain activity, heart rate, and the electrodermal response have been recorded. At the beginning of the recording, participants were asked to close and keep their eyes closed for 1 minute and after that to keep their eyes open, watching the black screen in front of them for 1 minute. This part of the recording was considered as baseline. For each product, the test protocol consisted of two phases lasting 15 seconds each: in the first one, participants had to look at the product, and in the second one, they interacted with the product, by freely manipulating it. After each phase, the experimenter asked the participant to rate the extent to which they appreciated the interaction with the product on a scale from −5 to +5. The protocol then restarted with another item. Products have been randomly presented among participants. A subsample of 13 subjects performed a further phase: they also interacted with the product before the visual exploration, with the eyes closed.

*Experiment 1:* 19 healthy participants (10M, average age = 24.94 ± 3.40, min = 21 years old and max = 31 years old) have been enrolled in the study. The selected products were (i) two Daily Food items (one representative of a Major Brand and one representative of a Private Label) and (ii) two Comfort Food items (one representative of a Foreign product and one of a Local product). The phases of interaction with the product lasted 15 seconds each and were the visual exploration (VE) and the visual and tactile exploration (VTE) phases.

Table 1 shows the examples of products belonging to each experimental category.

*Experiment 2*: 13 healthy participants (5 males, average age = 27.23 ± 7.30, min = 21 years old and max = 44 years old) have been enrolled in the study. For this experiment, four different Comfort Foods (e.g., chocolate bars) and four different Daily Foods (e.g., rice) have been chosen. For each category of food, 2 Local products and 2 Foreign products have been selected: both Local and Foreign products belonged to either the Major Brand or the Private Label categories (Table 1).

The protocol was divided into three phases: tactile exploration (TE) with closed eyes (15 s), visual exploration (VE) (15 s), and visual and tactile exploration (VTE) (15 s). The aim of this experiment was to investigate how the aesthetic design, the familiarity, and the cross-perception influence the impression of a product as a variation of cognitive and emotional variables.

### 2.1. EEG Recordings and Signal Processing

The EEG activity was recorded using ten electrodes (Fpz, Fp1, Fp2, AFz, AF3, AF4, AF5, AF6, AF7, and AF8) placed on an EEG frontal headband by means of a portable 21-channel system (BEmicro, EBneuro, Italy). Although the system allowed to record up to 21 channels, a ready-made headband with ten electrodes placed over the prefrontal and frontal cortex, since (i) only this cortical area (prefrontal and frontal) was of interest in the present study, and (ii) the headband reduced the system's invasiveness and increased the comfort of the participant when compared with traditional EEG caps. The reference and the ground electrodes have been placed, respectively, on the left and right earlobes. The signals have been acquired at a sampling rate of 256 Hz, and the impedances were kept below 10 kΩ. After the acquisition phase, the raw EEG signal has been digitally preprocessed by using the EEGLAB [46] Matlab toolbox. Firstly, a notch filter (50 Hz) was applied in order to reject the main current interference. Secondly, the gathered signal has been band-pass filtered by a fifth-order Butterworth filter ([2/30] Hz), in order to reject the continuous component as well as high-frequency interferences, such as muscular artefacts. Then, the independent component analysis (ICA), in particular the SOBI algorithm [47], has been applied to EEG data in order to identify and remove the component related to eye blinks and eye movements, since their contribution overlaps the EEG bands of interest [48]. The component (always one of ten) has been manually selected and removed, and after that the EEG signal has been reconstructed. Furthermore, in order to clean the EEG signal as much as possible, after these conservative steps (until now no EEG data has been lost), the EEG signal segments still affected by artefacts have been automatically detected and rejected. In particular, the EEG signal was segmented in 1-second-long epochs, shifted of 0.5 seconds. For each epoch, three automatic methods implemented in Matlab and based on “acceptability values” (in parentheses) have been used: the threshold criterion (±100 *μ*V), the trend estimation (±10 *μ*V/s, with *ρ* = 0.3), and the sample-to-sample difference (±25 *μ*V). If for a specific epoch at least one criterion was not respected, that epoch was labelled as “artefact.” Thus, the EEG epochs marked as “artefacts” have been removed with the aim to have a clean EEG dataset [30, 31].

Finally, in order to take into account any subjective difference in terms of brain rhythms, for each subject the individual alpha frequency (IAF) was computed on the 60-second-long closed eyes segment, recorded at the beginning of the experimental task. It consists in the individual power spectrum peak within the conventional alpha domain [7/12] (Hz) [43], in order to define the EEG bands of interest according to the method suggested in the current scientific literature (i.e., each band is defined as “IAF ± *x*,” where IAF is the individual alpha frequency, in Hertz, and *x* an integer in the frequency domain) [25]. Consequently, the EEG signal of each channel for each subject was filtered, by using a fifth-order Butterworth filter, in own alpha [IAF − 4/IAF + 2 Hz] and theta bands [IAF − 6/IAF − 4 Hz] [25], since they represent the more interesting bands for the purpose of the present study.

To compute the activity of the cortical areas of interest in a specific frequency band, the global field power (GFP) was then computed. This is a measurement introduced by Lehmann and Michel [49] in 1990 to summarize the synchronization level of the brain activity over the scalp surface. GFP is computed from a specific set of electrodes (the set depends on the involved brain area; in the following, it will be specified for each index) by performing the sum of squared values of EEG potential at each electrode, averaged for the number of involved electrodes, resulting in a time-varying waveform related to the increase or decrease of the global power in the analysed EEG. The GFP formula is presented as follows:

(1) $$\begin{array}{c} \text{GPF}=\frac{1}{N}\overset{N}{\underset{i=1}{\sum }}x_{\vartheta_{i}}{\left(t\right)}^{2}, \end{array}$$

where *ϑ* is the considered EEG band, frontal is the considered cortical area, *N* is the number of electrodes included in the area of interest (in this example, the frontal area), and *i* is the electrodes' index.

The formula defining the frontal alpha asymmetry (Approach Withdrawal (AW)) index is as follows:

(2) $$\begin{array}{c} \text{AW}=\text{GFP}\alpha \text{\_right}-\text{GFP}\alpha \text{\_left,} \end{array}$$

where the GFP*α*_right and GFP*α*_left stand for the GFP calculated among right (Fp2, AF4, AF8, and AF6) and left (Fp1, AF3, AF5, and AF7) electrodes, respectively, in the alpha (*α*) band. Higher frontal alpha asymmetry values, reported by the participants, stood for an approach motivation toward the stimulus, while lower frontal alpha asymmetry values stood for a withdrawal motivation [22, 23, 50, 51].

It may be argued that the tactile exploration, being characterized by the closed eyes condition, would be affected by an increase in the alpha range in comparison with both the visual exploration and visual and tactile exploration phases. However, the AW Index was calculated as the difference in the frontal alpha activity between the two hemispheres; therefore, alpha increase would not interfere with the estimation of the index.

To evaluate the mental effort/processing, EEG activity in the theta band of all the frontal electrodes has been considered for the GFP computation. An increase in the frontal theta (i.e., mental effort) would imply an increase in the task difficulty [33].

The AW Index and the Effort Index were estimated for each second and then normalized with respect to the index of the baseline (1 minute of open eyes).

It has been repeatedly evidenced in the literature, thanks to the application to several kinds of stimuli [29, 50, 52], that the frontal cortex constitutes an area of interest for the analysis of the approach or withdrawal attitude and the frontal theta as index of cerebral effort and processing.

The normalized AW and Effort values have been averaged along the whole duration of selected tasks for each subject: visual exploration (15 sec), tactile exploration (15 sec), and visual and tactile exploration (15 sec).

### 2.2. HR and EDR Recordings and Signal Processing

Electrodermal responses (EDR) and heart rate (HR) were recorded with a sampling rate of 64 Hz through a Shimmer (Shimmer Sensing, Ireland) system applied to the nondominant hand of the subject. More in detail, the cardiac activity was recorded by recording a Photoplethysmogram (PPG) (i.e., a pulse oximeter which illuminates the skin and measures changes in light absorption due to the heart pump, at the thumb). In order to obtain the HR signal from the PPG, the Pan-Tompkins algorithm has been used [53]. The constant voltage method (0.5 V) was employed for the acquisition of the skin conductance. The electrodes were placed on the palmar side of the middle phalanges of the second and third fingers, on the nondominant hand of the participants, according to published procedures [54]. Employing the LEDAlab software [55], the tonic component of the skin conductance (Skin Conductance Level, SCL) was estimated. The circumplex model of affect plane was adopted to collapse information about a stimulus deriving from SCL and HR[38,56]. In this model, the *x* axis reported the HR values, reflecting the valence dimension of a stimulus, while the *y* axis reported the SCL values, mirroring the arousal dimension of a stimulus [13]. Adopting this theoretical framework, it was possible to obtain a monodimensional variable, named the Emotional Index (EI), providing information concerning the emotional status of a participant, as defined in previous studies [39]. The EI results interpretation predict that higher values would mirror a more positive and engaging emotion experienced by the subject, and vice versa.

In the same way as the Cerebral Indexes, the Emotional Index value was estimated for each second and then standardized on the basis of the baseline (1 minute of open eyes).

### 2.3. Statistical Analysis

ANOVA was performed for each index (AW, Effort, and Emotional) for the factor phase with three levels (tactile exploration (TE), visual exploration (VE), and visual and tactile exploration (VTE)), singularly for each of the following means categories:

- Comfort Food versus Daily Food
- Major Brand versus Private Label
- Foreign versus Local

For all the indexes (AW, Effort, and Emotional), paired *t*-test was performed singularly for each phase (tactile exploration (TE), visual exploration (VE), and visual and tactile exploration (VTE)), in order to compare the means of

- Comfort Food versus Daily Food
- Major Brand versus Private Label
- Foreign versus Local

For the Major Brand versus Private Label comparison, we included only Local products in the analysis, due to the lack of knowledge of the Foreign labels that could bias the perception of the Brand level.

Since the correspondence between the phases, data from Experiments 1 and 2 were collapsed for the analyses performed on the VE and VTE phases (therefore, VE and VTE phases included 32 participants), while for the TE phase, only data from Experiment 2 were available (therefore including data from 13 participants).

In addition, Fisher's exact test was performed in order to analyse the distribution of the behavioural categorical data (e.g., the declared knowledge of the product).

## 3. Results and Discussion

### 3.1. Results

#### 3.1.1. Comfort Food versus Daily Food Comparison

**Figure 1:**
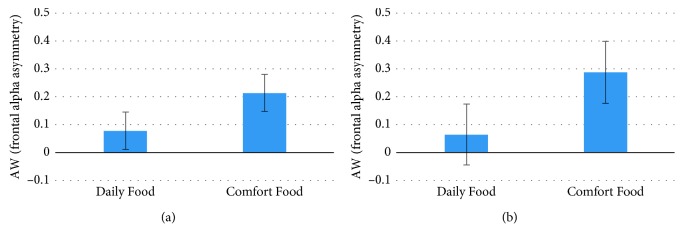
The graphs represent the statistical results for the AW Index values concerning the comparison between the Daily Food and Comfort Food categories. (a) Mean AW values during the visual exploration phase (statistically significant difference *p*=0.031) and (b) mean AW values during the visual and tactile exploration phase (statistically significant difference *p*=0.027). Error bars represent standard error.

**Figure 2:**
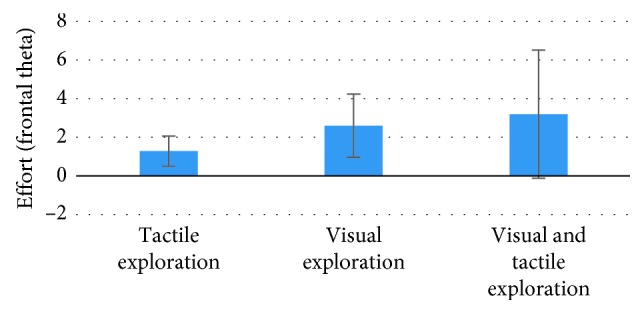
The graph represents the statistical results for the Effort Index values concerning the comparison among the experimental phases (visual exploration, tactile exploration, and visual and tactile exploration) (phase effect *p*=0.003). The post hoc analysis evidenced how the tactile exploration phase presented lower effort values in comparison with both the visual exploration (*p*=0.016) and the visual and tactile exploration phases (*p*=0.001). Error bars represent standard error.

The comparison between these two categories of products showed a higher tendency of approach in response to Comfort Food than Daily Food as evaluated by the AW Index both in the visual exploration phase (VE) (*p*=0.031, *t* = −2.26, Cohen's *d* = 0.297, *df* = 26) and in the visual and tactile exploration (VTE) phase (*p*=0.027, *t* = −2.342, Cohen's *d* = 0.537, *df* = 26) (Figure 1). The significance was not found for the AW Index during the tactile exploration (TE) phase (*p*=0.443,*t* = 0.794). Concerning the Effort Index, a statistically significant effect of the phase has been found (*p*=0.003, *F*(2, 22) = 7.516), with lower values reported for the TE in comparison with both the VTE (*p*=0.001) and VE (*p*=0.016) phases (Figure 2). The same ANOVA performed on AW and EI data considering the factor phase (TE, VE, and VTE) did not provide statistically significant results.

#### 3.1.2. Major Brand versus Private Label Comparison

The comparison between the Major Brand and the Private Label products categories highlighted higher positive rating values for the Major Brand than for the Private Label products belonging to the Comfort Food category during the VE (*p*=0.001, *t* = −4.535, Cohen's *d* = 1.865, *df* = 11) and VTE (*p*=0.001, *t* = −4.371, Cohen's *d* = 1.780, *df* = 11) phases, but not during the TE phase (*p*=0.919, *t* = 0.104). On the contrary, concerning the Daily Food category, the Private Label obtained higher rating values than the Major Brand category in the TE (*p*=0.019, *t* = 2.749, Cohen's *d* = 0.963,*df* = 11) and in the VTE (*p*=0.018, *t* = 2.768, Cohen's *d* = 1.128, *df* = 11) phases but not in the VE phase (*p*=0.186, *t* = 1.412). In addition, the Fisher exact test evidenced higher values of product recognition for the Major Brand than for the Private Label products concerning both the Comfort Food (*p*=0.036) and the Daily Food (*p*=0.003) categories. Focusing on the rated products (data from Experiment 2), a higher AW value for the Private Label in comparison with the Major Brand products during the VTE phase has also been found (*p*=0.018, *t* = −2.788, Cohen's *d* = 0.364,*df* = 11). Other paired *t*-test and ANOVA performed on AW, Effort, and Emotional Indexes did not report any statistical significance.

#### 3.1.3. Foreign versus Local Products Comparison

**Figure 3:**
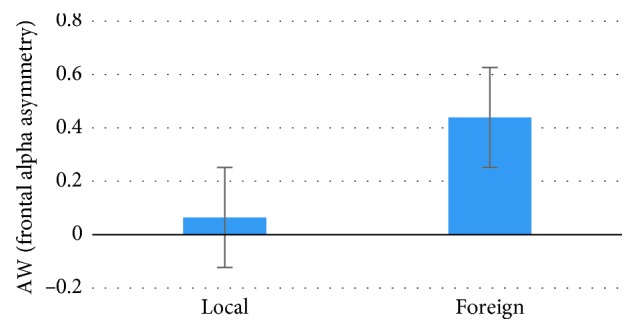
The graph represents the statistical results for the AW Index values concerning the comparison between the Local and Foreign products categories. Error bars represent standard error.

**Figure 4:**
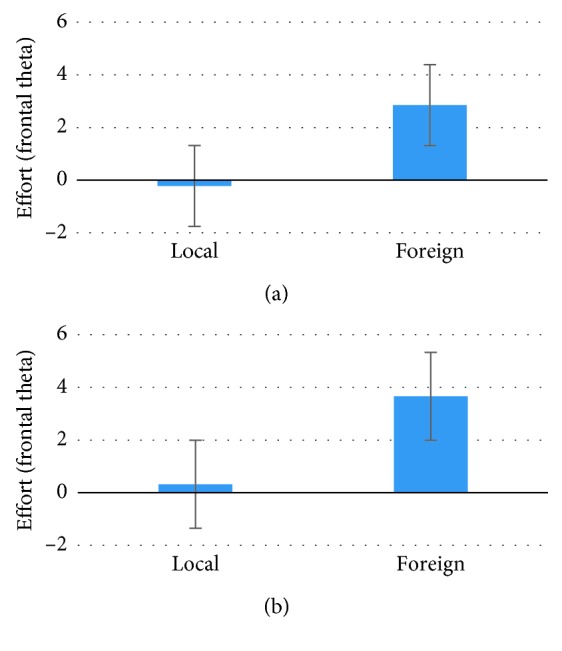
The graph represents the statistical results for the Effort Index values concerning the comparison between the Local and Foreign products categories. (a) represents mean Effort Index values during the visual exploration phase (statistically significant difference *p*=0.001) and (b) represents mean Effort Index values during the visual and tactile exploration phase (statistically significant difference *p*=0.003). Error bars represent standard error.

The analysis demonstrated higher AW Index values for the Foreign products in comparison with the Local ones, supporting a higher tendency of approach toward the former products only during the VTE phase (*p*=0.034, *t* = −2.224, Cohen's *d* = 0.518, *df* = 29) (Figure 3), but not in the TE (*p*=0.368, *t* = 0.938) and VE (*p*=0.169, *t* = −1.409) phases. Similarly, considering the Effort Index, an increased effort value for the Foreign products in comparison with the Local ones in the same VTE phase has been shown (*p*=0.003, *t* = −3.232, Cohen's *d* = 0.834, *df* = 29) (Figure 4(b)), and also in the VE phase (*p* < 0.001, *t* = −3.666, Cohen's *d* = 0.965,*df* = 29) (Figure 4(a)), but not in the TE phase (*p*=0.266, *t* = 0.171). Finally, concerning the EI, higher values were found in response to the Foreign products during the TE phase (*p*=0.010, *t* = −3.049, Cohen's *d* = 0.483, *df* = 12), but not in the VE (*p*=0.508, *t* = −0.673) and VTE (*p*=0.394, *t* = −0.868) phases. ANOVA on the comparison among phases (TE, VE, and VTE) did not evidence any statistical significance for any of the indexes.

## 4. Discussion

The EEG results supported the hypothesis 1 that the Comfort Food category elicited a higher tendency of approach (higher AW values) than the Daily Food category, as also found in previous evidences, reporting higher AW values for sweet foods, and in particular for chocolate, in comparison with other foods [57]. Moreover, it is interesting to note that this pattern was found for the VE and VTE phases but not for the TE phase, therefore suggesting that the visual modality was prominent over the touch for the hedonic evaluation of the tested products. This result was also supported by the evidence that there was a lower processing (as indexed by the mental effort values) during the TE phase in comparison with both the VE and VTE phases.

The lack of statistically significant differences in the comparison between Major Brand and Private Label products, for what concerns the investigated neurometric indices, could be explained by the strong balance and correspondence between analogous products selected in the different categories. On the contrary, the identified differences between Major Brand and Private Label in the behavioural results displayed how the Major Brand products were the most recognized by participants. In addition, the higher verbally declared pleasantness reported for the Major Brand Comfort Food in comparison with the Private Label Comfort Food in correspondence of the VE and VTE phases suggest that for Comfort Food, the visual modality is prominent in the evaluation of Comfort Food quality. On the contrary, for the Daily Food, we found higher rated pleasantness for the Private Label Daily Food in comparison with the Major Brand Daily Food during the TE and VTE phases (both involving the tactile modality). This could be explained by the characteristics of the package, since the Private Label Daily Food was a plastic bag that allowed participants to “feel” the rice seeds when touching, while the Major Brand Daily Food was constituted by a carton box that did not confer direct information about the products inside.

Results on the comparison between Foreign and Local products verified hypothesis 2 and were consistent with Mandler's theory [45], which demonstrated that a complex stimulus, so one requesting a high information processing, raised more interest than a simple one generating an approach toward itself (as shown by the highest AW value).

Mental effort results showed that the sample had higher effort value for the foreign product, during both the VE and VTE phases, probably because of not having familiarity with them. These results could be caused by the novelty of the visual features of the Foreign products, which requested higher cognitive activity to process the information. In fact, when the stimuli do not have sufficient attributes which call back a preexisting cognitive model, people spend more effort for acquiring the new information and for creating a new cognitive model [58]. In this case, because of the lack of familiarity with the foreign products, the sample spent more cognitive effort for processing information during the interaction with them. Interestingly, during the TE phase, we did not find any statistical difference between Foreign and Local products, possibly explained by the fact that the corresponding goods selected belonging to the Foreign and the Local categories were extremely similar from a haptic point of view.

Concerning the AW Index, the value reported for Foreign products was higher only during the VTE phase, maybe because of the presence of more appealing design and informative contents on the back of the packages; for instance, the Foreign Major Brand Daily Food product presented a naturalistic scenario on the back, instead of some extremely predictable information, as was the case on the Local Major Brand Daily Food one.

Furthermore, studies demonstrated that the novelty of a product, the difficulty in its categorization when compared to preexisting mental images of that product, and the incongruity between the novel product and the experienced known products belonging to the same category mirror to cognitive patterns underlying an increased curiosity toward the product and an increased tendency of approach toward it. In particular, Olivero and Russo's studies [59] demonstrated that when a product matched exactly the preexisting mental patterns, it loses its capacity to attract, causing boredom and avoidance from itself: the novelty stimulates the curiosity, interest, and the approach toward the product, especially for people called “*Sensation Seekers*,” who are easily bored without high levels of stimulation.

## 5. Conclusions

In summary, the results of the present study could be summarized as follows:

HP1 was confirmed, since different cognitive and emotional reactions have been obtained, in particular during the VTE phase in response to Comfort Food.

HP2 was also demonstrated, since the neuroelectrical indexes for the Foreign products showed higher values of Effort Index during all the phases in which the visual modality was involved and a higher approach tendency (AW Index) toward them during the free manipulation of such products.

HP3 was not confirmed; in fact, we did not detect any statistically significant difference in the neurophysiological reaction to the Major Brand and the Private Label items; however, this could be explained by the strong analogy and balance among the selected items.

The present study has the obvious limitations of the number of the food packaging products tested, although the sample size of participants was sufficient to reveal significant statistical effects. The future studies should preferably more focused address the study of different food packages, constituted by different materials (e.g., tetrapack bricks). Nevertheless, the study demonstrated the possibility to investigate the cerebral and emotional reactions of a group of normal subjects and potential buyer to the visual and tactile exploration of food products with a number of neurometric indexes.

## Figures and Tables

**Table 1:** 

Comfort Food samples	Local products	Foreign products

Major Brand	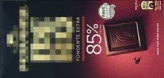	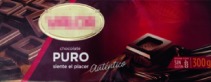
Private Label	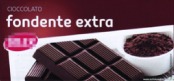	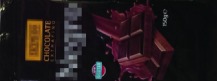

Daily Food samples	Local products	Foreign products

Major Brand	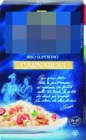	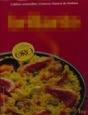
Private Label	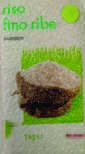	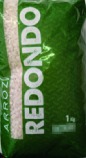

## Data Availability

The data used to support the study could be obtained by sending an email to Prof. Babiloni (fabio.babiloni@uniroma1.it).
